# On the changes of the intention to leave the parental home during the COVID-19 pandemic: a comparison among five European countries

**DOI:** 10.1186/s41118-021-00117-7

**Published:** 2021-06-23

**Authors:** Francesca Luppi, Alessandro Rosina, Emiliano Sironi

**Affiliations:** Deparment of Statistics, Università Cattolica del Sacro Cuore, Largo Gemelli 1, 20123 Milan, Italy

**Keywords:** COVID-19, Leaving parental home, Intentions

## Abstract

With the spread of the SARS-CoV-2 pandemic in Europe during the first months of 2020, most of the governments imposed restrictive measures to people mobility and physical distance (the lockdown), which severely impacted on the economic activities and performance of many countries. Thus, the health emergency turned rapidly into in an economic crisis. The COVID-19 crisis in Europe increased the uncertainty about the economic recovery and the end of health emergency. This situation is supposed to have conditioned individuals’ life course path with the effect of inducing people to postpone or to abandon many life plans.

This paper aims to explore and describe whether the rise of health emergency due to the COVID-19 has delayed or vanished young people’s intention to leave the parental home, in order to establish their own household, during 2020 in five European countries: Italy, Germany, France, Spain and the UK. Using data from an international survey from the “Youth Project”, carried out by the Toniolo Institute of Advanced Studies, this paper implements generalized logistic models for ordinal dependent variables to investigate the factors associated with a possible revision of the choice of leaving the parental home for a representative sample of 6000 respondents aged 18 to 34, interviewed between March and April 2020. In particular, we compare the effect of the occupational condition and the perceived income and employment vulnerability on the chance of confirmation, postponement or abandonment of the pre-pandemic plan across the five selected European countries.

Results show that Italy, Spain and the UK are the countries with the highest probability of a downward revision of the intentions of leaving the nest. Especially in these countries, having negative expectations about changes in the individual’s and family’s future income is associated with the choice of abandoning the purpose of leaving the parental home. However, the vulnerability of the category of temporary workers particularly arises in Southern European countries: young people with precarious jobs seem to be the most prone to negatively revise their intentions of leaving, even compared with those not working.

## Introduction

Economic recessions tend to prolong the time needed for young people to make their transition to adulthood. The 2008 Great Recession has largely shown the mechanisms at work: because economic crises especially reduce youth employment opportunities (Bell & Blanchflower, 2011; Schoon & Bynner, 2017); the pre-existing tendency towards prolonged education participation and precarious occupations has been accentuated. This process resulted, for many young people, into a postponement of some life events such as reaching housing and economic independence from the family of origin. These effects have been especially evident among Southern European countries (Aassve, Arpino, and Billari, 2013; Aassve, Cottini, and Vitali, 2013; Ahn & Sánchez-Marcos, 2017; Sironi, 2018), where the effect of the economic recession summed up with an unfavourable labour market situation, scarce welfare state provisions and a struggling economic system. In this context of less-protective transition regimes (i.e., from youth to adulthood), the result has been a strong increase of the youth unemployment rate, the spread of involuntary temporary occupations and the enlargement of the subgroup of young individuals neither in employment nor in education or training (NEET) (e.g., Schoon & Bynner, 2019). All these conditions have been found related with a lower propensity to undertake the traditional steps toward the transition to adulthood (e.g., Bryson & Harvey, 2000; Golsch, 2003; Rosina, Micheli, & Mazzucco, 2007; Sironi & Rosina,^ 2015^; Vignoli, Tocchioni, & Salvini, 2016).

The COVID-19 health crisis, which picked in Europe with the lockdown restrictions implemented in most of the European countries between February and March 2020, had suddenly translated into a severe global economic recession. Because of the previous considerations, even though we cannot assimilate the COVID-crisis with the 2008 Great Recession, we can expect that this economic downturn will strongly hit—again—the young population, threatening their life plans. Overall in Europe, first evidence from the 2020 labour market trends shows that the COVID-19-related recession has increased the unemployment rate more among the young population (i.e., 18–34 years old), women, lower educated and self-employed workers (Eurofound, 2020). Moreover, in Western Europe, the likelihood of losing a job during the 2020 was systematically higher in Southern European countries compared with the others, and among workers with temporary and precarious occupations (Eurofound, 2020). In the same countries, also the perception about how things will go in the future seems to be more pessimistic: in particular, the proportion of those expecting the financial situation will get worse in the future is higher in Spain, Italy and Greece compared with the other Western European countries (Eurofound, 2020).

Young individuals have higher chances to be employed with temporary contracts and as casual workers, which makes their position in the labour market extremely vulnerable to unemployment especially in Southern European countries (Barbieri & Scherer, 2009; Gebel & Giesecke, 2016; Golsch, 2003). As they face a higher risk of becoming unemployed—compared with workers more embedded into the labour market—also their financial autonomy from the family of origins might be compromised. Even though they do not directly experience a job loss, they might perceive a great uncertainty about their future occupational position and income, which would probably impact their life choices (Bryson & Harvey, 2000). Therefore, the decision to exit the parental home—which requires reaching some economic independence—might be easily compromised during an economic recession period.

The aim of the present study is to describe the variation in the intention of leaving the parental home—for own household formation—because of the COVID-crisis, among a set of five European countries (i.e., Italy, France, Germany, Spain and the UK). In particular, we investigate whether previous individual’s occupational condition and the expected effect of the crisis on the individual’s and family’s income are associated with the decision of withdrawing or postponing the plan of acquiring a housing independence and whether this relationship changes across the selected countries. The analyses have been conducted on data coming from an international survey on representative samples of the young population (18–34) of the five European countries. The interviews have been conducted between March 27 and April 7, 2020, when the lockdown restrictions had been already adopted in most of the considered countries.

## Background

Leaving the parental home is one of the first steps in the transition to adulthood. This typically happens for reasons such as studying in another city/country, because the individual reaches an economic independence from the family of origin, or because of starting a cohabitation with a partner (Billari, Philipov, & Baizán, 2001; De Jong Gierveld, Liefbroer, & Beekink, 1991). Historically, the age of leaving the family of origin has grown over time, particularly since the late sixties of the last Century, due to the institutional and values changes that took place in Western societies in that period (Aassve, Billari, Mazzuco, & Ongaro, 2002; Furstenberg Jr., 2010; Lesthaeghe & Van de Kaa, 1986; Settersten Jr., Furstenberg, & Rumbaut, 2008; Sironi & Furstenberg, 2012). The increased family investment in children, the prolonged time spent by children in the education system, the desire of finding a job that matches their qualification, and the increased well-being of the families of origin are among the factors that have contributed to postpone the “right” time of leaving the parental home (Buchmann & Kriesi, 2011; Sironi, 2015). Almost unavoidable, it is the prerequisite of having acquired the economic self-sufficiency or getting access to the financial support from the family of origin or from a partner (Aassve et al., 2002; Goldscheider & Goldscheider, 1993; Iacovu, 2010). However, the effect of employment and income conditions is not always clearly related with the chance of leaving the parental home across European countries (Aassve et al., 2002), even though young people tend to stay longer in the parental home in case leaving would increase their poverty risk (Aassve, Davia, Iacovu, & Mazzucco, 2007). These cross-country differences are due to the fact that the timing and the reasons for leaving the parental home varies across European countries (Billari et al., 2001). Hence, for example, in the European context, Scandinavians tend to leave the parental home earlier, while in Southern Europe, the average age to exit the family of origins is the highest (Billari, 2004). About the reasons for leaving the parental home, in Southern European countries, this transition is often linked to marriage (Baizan, 2001; Billari, Castiglioni, Castro Martin, Michielin, & Ongaro, 2002; Cavalli & Galland, 1996; Holdsworth, 2000; Rusconi, 2004); in French, Germany and Northern Europe, leaving the parental home is more often associated with (short) time spent living alone or entering forms of union other than marriage (Cavalli & Galland, 1996; Galland, 1997; Kynčilová, 2009; Luetzelberger, 2014); in the UK the fact that students in tertiary education are often moving to in-campus accommodations (Aassve et al., 2002), together with the early transition from school to work, it pushes young individuals out of the family of origin, while the family formation is often delayed (Berrington, 2001; Cavalli & Galland, 1996; Holdsworth, 2000).

Typically, in the literature, the intentions and the behaviours of leaving the family of origin is linked also to some macro-level socioeconomic and cultural factors (Buchmann & Kriesi, 2011), such as the structure of the labour market, the dominant values system and the type of welfare state.

As a result of the globalization process, the structure of the labour market has changed dramatically since the last 30 years, by increasing occupational uncertainty at every stage of the work career, but especially for those entering the labour market for the first time (Mills & Blossfeld, 2003). Young people, in fact, usually experience greater difficulties in getting a job and higher instability of their positions and contracts if compared with those with longer working histories (Blanchflower & Freeman, 2000; Breen, 2005; Brzinsky-Fay, 2007; Gangl, 2002; Scherer, 2005). The labour market condition has a strong power in explaining the different timing of leaving the parental home across countries (Aassve et al., 2002), because having a stable job and/or experiencing low risk of unemployment is a precondition for reaching a financial autonomy. On the contrary, a context where young people are at high risk of unemployment discourages from leaving the parental home. This mechanism at least partially explains the lower propensity of young individuals in Southern Europe to make this step towards adulthood: Italy, Greece and Spain, in fact, are the Western European countries with the highest proportion of NEET (i.e., young people not Employed neither in Education nor Training) (in 2019, among those aged 15 to 34, the percentage of NEETs in Italy was 23.8%, in Greece 20.1% and in Spain 16%, while the EU28 average was 13.6. Source: Eurostat). Their incidence has especially risen as effect of the 2008 Great Recession, which increased youth unemployment through hitting temporary contracts, largely widespread among young, low-skilled workers (Lin, Edvinsson, Chen, & Beding, 2013; Sironi and Rosina, 2015; Mascherini & Ledermaier, 2016). However, the NEET population is not homogeneous, and its composition varies across countries and labour markets (Mascherini & Ledermaier, 2016). In particular, vulnerable NEETs (those with longer periods of unemployment, higher chance of being discouraged or not actively looking for a job, also due to family responsibilities) are more common in Southern European countries, where the structural weaknesses of the labour market and the economic stagnation reduce the chance of finding a (new) job. On the contrary, non-vulnerable NEETs (i.e., short-term unemployed persons and opportunity seekers) are more present where the labour market and the economic system are in good health (as in France and the UK, where the proportion of NEET was respectively 14% and 11.7% in 2019, but most of them were short-term NEET. Source: Eurostat database; Mascherini & Ledermaier, 2016). In liberal countries, such as the UK, the access to the labour market is somehow easier than in Southern European countries because of its better performance, but the occupational conditions are usually less stable than in other countries (Buchmann & Kriesi, 2011).

The welfare policies can act positively in reducing the uncertainty due to unfavourable conditions of the labour market or occupational instability, and thus supporting young people in their transition towards financial autonomy. It has been widely shown in the literature that the type of welfare state is linked to different timing and paths to adulthood (Buchmann & Kriesi, 2011; Sironi, 2015), with the most generous welfare systems effectively promoting the process of leaving the parental home. By referring to the Esping-Andersen typology (Esping-Andersen, 1990), in the cluster of Southern European countries, the transition to adulthood happens later in life if compared with countries characterized by other types of welfare regimes (Aassve et al., 2002; Aassve, Iacovou, & Mencarini, 2006; Aassve & Lappegård, 2009; Billari, 2004; Billari et al., 2001; Esping-Andersen, 1990; Mills et al., 2008; Mulder, Clark, & Wagner, 2002). Here, in the Italian case in particular, the welfare system is not particularly generous towards young people (Barbieri, 2011; Rosina et al., 2007), and commonly, the family network balances the low provision of support provided by the welfare state (Mayer, 2001; Trifiletti, 1999). Also liberal welfare states (such as the UK) are characterized by low levels of public support; however, in this case the prevalence of weak ties in the family, while promoting young people’s autonomy, does not guarantee an informal safety net (Buchmann & Kriesi, 2011). Consequently, while young people in familialistic countries (i.e., in Southern Europe) tend to minimize their income risk by relying on family support and postponing the leaving from the parental home (Blossfeld, Klijzing, Mills, & Kurz, 2005), this is not a common path in liberal welfare regimes. Obviously, in familialistic countries, the family support is conditional to the economic and social situation of the family of origin, and in particular to its possibility to financially sustain child’s decision of reaching a housing independency (De Jong Gierveld et al., 1991; Santarelli & Cottone, 2009). Finally, conservative welfare regimes (such as France and Germany) are somehow in the middle, enabling a smooth and quite stable labour market entry, while their still typical family orientation does not push young people’s autonomy (Buchmann & Kriesi, 2011).

The institutional setting conditions the transition to adulthood also through the values system, which is implicit in the everyday social interactions, especially in the family context. Parental attitudes and value orientation (Goldscheider & Goldscheider, 1989; Surkyn & Lesthaeghe, 2004) guide the evaluation of the preconditions and the timing for leaving the parental home. Cultural differences are wide across countries. As previously mentioned, in the Southern European countries, family relationships, and in particular, those between parents and children, are especially solid, because they often are the primary source of emotional and material support for young adults (Dalla Zuanna, 2001; Dalla Zuanna & Micheli, 2004). As already said, this special type of intra-family relationship derives also by the institutional setting—and the type of welfare state in particular—whose inefficiency in fostering young people economic independence is often compensated by the support provided by the family network. On the opposite side, in the UK, weak family ties push young people out of the parental home quite early in their life.

The economic cycle phase also matters; recessions, in fact, have the power to increase the difficulties in making the transition to adulthood (Kohler et al., 2002). This happens because young people are extremely financially vulnerable to economic crises (Aassve, Arpino, and Billari, 2013; Aassve, Cottini, and Vitali, 2013; Bell & Blanchflower, 2011; Cho & Newhouse, 2013; Grusky, Western, & Wimer, 2011; Hout, Levanon, & Cumberworth, 2011; Mínguez, 2017; O’Higgins, 2014; Sironi, 2018; Verick, 2009). Young people who were employed before the crisis are usually in sectors that are particularly affected by recessions (O’Higgins, 2014; Verick, 2009). Additionally, because employed young people are at the beginning of their work career, they usually rely on informal or temporary contracts, which are easily at risk of ending in recession periods (Marcus & Gavrilovic, 2010). Finally, those who did not enter the labour market yet face increased difficulties in finding a job, while if they finally succeed to get an occupation, it is usually very precarious (Sironi, 2018). Because recessions undermine the occupational stability, as the prospect and the real earning of young people, which are prerequisites for gaining a financial and housing autonomy, most of them postpone the steps towards the transition to adulthood (Aassve et al., 2002; Bell, Burtless, Gornick, & Smeeding, 2007; Furstenberg Jr., 2010; Iacovu, 2010). However, recessions do not hit the young population uniformly, as it may depend also on the type of welfare regime and values system. More protective and generous welfare regimes towards young generations (e.g., sustaining the transition from education to labour market, providing income and housing support, etc.) tend to reduce the economic uncertainty and vulnerability derived by the economic crises (Schoon & Bynner, 2019). In less protective welfare regimes, such as in Southern Europe, given the occupational and income instability due to the recession, young people may simply decide to prolong their stay in the parental home to avoid any additional income risks (Sironi, 2018).

## Data, method and variables

Data used in this study come from the “Youth Project”, a survey started in 2011 within the financial support and the partnership of the Toniolo Institute of Advanced Studies and CARIPLO Foundation and with IPSOS LTD as executive and technical partner. In 2020, an international survey promoted within the “Youth Project”, focuses on the consequences of the COVID-19 pandemic on the life course plans of a representative sample of the population of European young adults (people aged 18 to 34). A sample of 6000 European young adults participated in the research. Among them, 2000 come from Italy and 1000 come from each of the following European countries: the UK, Germany, France and Spain. The interviews have been conducted between March 27 and 31, 2020 in Italy and April 2 and 7, 2020 in the other countries. The survey was realized using casual stratified sampling and is a subsample of the “Online panel of Ipsos” (further detail on the sample are in Appendix, Table 7). Strata are defined using the following variables: gender, age, geographical origin (NUTS1 level), the size of municipality, education level, marital status, labour market condition (working or not, student or not). Hence, Strata are designed to reflect the population and so that the sample is representative of the country’s population after having corrected frequencies through a weighting procedure: the population distribution of reference for the computation of weights is according to data from Eurostat dating back to 2019. Sampling weights are computed as the ratio between the proportion in the population and the proportion of the sample of each stratum. The interviews were carried using the CAWI method (Computer-Aided Web Interviewing: the questionnaire is available online; the interviewee opens the webpage and automatically answers the questions that appear on the screen. More technical details are available at https://www.rapportogiovani.it/osservatorio/ and in the Technical Note included in the Youth Report 2021 (Istituto Toniolo, 2021).

Descriptive statistics about the socio-demographic characteristics of the interviewed individuals are reported in Table 1.

**Table 1:** Descriptive statistics of the total sample distribution

	Italy	UK	Germany	France	Spain
**Gender**					
Male	51.0%	50.6%	52.1%	49.6%	50.4%
Female	49.0%	49.4%	47.9%	50.4%	49.6%
**Age (in years)**					
18–24	31.9%	31.2%	30.3%	32.7%	30.2%
25–29	31.8%	34.4%	34.2%	32.3%	32.1%
30–34	36.3%	34.4%	35.5%	35.0%	37.7%
**Residential status**					
Already left parental home	51.1%	70.6%	76.5%	74.0%	56.0%
Never left parental home	48.9%	29.4%	23.5%	26.0%	44.0%
*Observations*	*2000*	*1000*	*1000*	*1000*	*1000*

Source: Youth project, International survey (2020)

The 2020 International survey includes a set of items exploring European young adults’ perspectives on their life plans for the same year (e.g., leaving the parental home, conceiving a child, getting married, started a cohabitation with the partner, moving to another country, changing the current job). This paper focuses on the decision of European young adults to leave the parental home during 2020 and on how they reconsidered the plan in light of the effects of the COVID-19 pandemic. Hence, the sample explored in the data analysis is restricted to the subset of individuals who live with their family of origin at the time of the interview (March/April 2020) and who were considering going to live on their own at the beginning of the year (January 2020).

As shown in Table 1, different patterns of living arrangements characterized the five sampled countries. With respect to the residential condition, in Italy, the percentage of young adults who still live within the family origin at the time of the interview is greater than in the other countries (48.9%), but similar to the Spanish case (44%). This result confirms the Southern European habit of leaving the nest later, at least if compared with the other countries considered in this study: in Germany, France, and the UK, in fact, the percentage of individuals living with the parents is lower than 30% in our sample. This gap in the proportion of those who have already achieved housing self-sufficiency across countries has been linked in the literature to the different occupational vulnerability experienced by the young workforce.

Table 2 shows the proportion of individuals in the sample who were considering going to live on their own before the beginning of COVID-19 emergency. The percentage of individuals with a clear plan of leaving the parental home in the pre-pandemic period is lower than the percentage of those who were intentioned but without a plan. Only apparently surprising is the results of Italy, who displays the highest rate of people intentioned to leave the nest. The result is related to what was discussed in Table 1: Italy is the country with the highest proportion of individuals still living with their parents (and having postponed in the past years).

**Table 2:** People that were considering going to live on their own in January 2020

	Italy	UK	Germany	France	Spain
People who were considering but not planning on it	22.5%	14.6%	20.6%	16.9%	15.9%
People who were planning on it	13.1%	11.6%	6.1%	15.9%	11.2%
*Observations*	*2000*	*1000*	*1000*	*1000*	*1000*

Source: Youth project, International survey (2020)

Results in Table 3 focus on the decision to confirm or to revise the intention of leaving the parental home, in light of the COVID-19 pandemic. In particular, the question asks “Now we’ll talk about your present and future plans. At the start of the year, just before the coronavirus outbreak, were you planning on one of the following events, to be completed by the end of 2020? Going to live on your own” with three possible answers: (1) “No”, (2) “I was considering it but hadn't made any plans”, (3) “Yes, I was planning on it”. To those answering (2) or (3), a further question has been asked: “Has the coronavirus emergency interfered with that plan in any way?”, with three alternative answers: (1) “No, the plan is still confirmed for 2020”, (2) “The plan is still happening but I had to postpone it” and (3) “I've abandoned that plan”. Therefore, the analyses have been conducted on the subsample of individuals that reported a positive retrospective intention of leaving the parental home in January 2020 (answers (2) and (3)), and exploring the chance of confirming, postponing or abandoning the original plan. Overall, the COVID-19 emergency has had a strong impact in depressing the purposes of the interviewed individuals: in all the European countries the percentage of those who postponed or abandoned the plan of leaving the parental home exceed sharply the percentage of those who confirmed their plan.

**Table 3:** People who were reconsidering going to live on their own in light of COVID-19 pandemic (answers conditioned on positive answers to the item in Table 2)

	Italy	UK	Germany	France	Spain
People who confirm the plan, despite COVID-19 pandemic	19.7%	25.1%	30.6%	31.7%	19.4%
People who declared that plan is still happening, but it was postponed	45.6%	50.7%	46.4%	55.3%	51.4%
People who abandoned that plan	34.7%	24.2%	23.0%	13.0%	29.2%
*Observations*	*733*	*240*	*257*	*334*	*282*

Source: Youth project, International survey (2020)

Although the pandemic had a generalized impact of delaying or denying the choice of young adults of going to live on their own, some specificities at the country level arise. In particular, Italy performs worse with regard to the other countries, showing the highest proportion of young adults who revised the intention of exiting from the parental home: 45.6% of the respondents decided to postpone their plans, while 34.7% of them totally abandoned the idea. Coherently with the expectations, in this framework, Spain is the runner-up in the ranking of revised intentions: 29.2% of respondents renounced to go living on their own.

Nevertheless, the economic instability and the sense of uncertainty due to the spread of COVID-19 also hit the young people’s plans in Germany and the UK. In France, the effect of the pandemic seems to be milder, even though those who postponed the plan counterbalance those who abandoned it.

Hence, the dependent variable of the model is the one presented in Table 3, with three possible levels of responses *j* hierarchically ordered from the most optimistic intentions of leaving the parental home (*j = 1*) to the less optimistic one (*j = 3*), which overlaps with the choice of a complete renounce of the intention to leave. In more detail, the following is the pool of available levels for variable *j*:

- Confirmation of the plan [of going to live outside the family of origin] (*j = 1*)
- Postponement of the plan [that is formally confirmed, but delayed for what concerning the date of departure] (*j = 2*)
- Abandonment (suspended *sine die*) of the plan to reach residential autonomy. Two different model specifications are used to model the responses of the outcome variable (*j = 3*)

Because of the three ordered levels of the outcome, the ordered logistic model should be the gold standard approach. Nevertheless, to produce consistent estimates for the parameters, the validity of the parallel line conditions must be assumed. The parallel regression assumption states that the coefficients that describe the odds of being in the highest category vs. all lower categories of the response variable are the same as those that describe the odds between the second highest category and all lower responses, and so on for the third highest category vs. all the lower ones. When the parallel line assumption holds, an ordinal logistic regression is preferrable to a multinomial one. When the assumption is violated, we should replace the ordinal logit with a multinomial one. If there is a violation of the parallel line assumption only for a limited set of covariates, the estimate of a multinomial regression on the whole set of parameters would not be a parsimonious choice.

In this framework, we adopt a more flexible model which provides unbiased and consistent estimates even if the parallel line assumption does not hold, which is well known in literature as the generalized logistics regression (Williams, 2016); this model allows performing different strategies to treat variables that fail the parallel line assumptions and those not violating that condition. Let *Y*_*i*_ be the outcome for the individual *i*: under the ordinality assumption of the response outcome in three different levels (*j = 1, 2, 3*), the model assumes the following form:

$$ \Pr \left({Y}_i\le j|{X}_i\right)=F\left({X}_i\beta \right)=\frac{1}{1+\exp \left({\alpha}_j+{X}_i\beta \right)} $$

The parallel line assumption states that parameters *β* should not change for different categories *j*. If it does not happen, then the size of the coefficients of some explanatory variables depends on the cut-off points of the dependent variable.

A Brant test (Brant, 1990) has been implemented to verify whether the parallel line assumption holds or not, showing that in every model for at least one variable parallel line is violated^1^. With respect to those coefficients that do not pass the Brant test and for which the parallel line assumption condition does not hold, two different coefficients are estimated for the following specification: one deriving from a first model with category 1 (confirmation of plans) versus categories 2 and 3 (postponement or abandonment) joined together as dependent variable and a second one deriving from a model with categories 1 and 2 (confirmation or postponement) joined together versus category 3 (abandonment); with respect to those coefficients that pass the Brant test, a unique coefficient has been reported for the two regressions. In practice, when the coefficients in the adjacent columns (within the same model) are the same, then the parallel line assumption holds. When they differ, it means that the parallel line assumption is violated.

The main covariates are represented by the employment status of the respondents and his/her perception of their future income and occupational condition. These variables should catch the economic vulnerability experienced at the beginning of the COVID crisis.

The *employment status* is strongly related with the available economic resources. We consider five categories: (1) students, (2) NEETs, (3) temporary workers (employees with a fixed-term job), (4) permanent employees (employees without a contract expiration date), and (5) self-employed (a category including young entrepreneurs and freelancers, which has been chosen as the reference category).

The *perceived future income and job conditions* is a variable which explores respondents’ expectation about their future job and the future income of their family. The items used for building the variable collect the answers to the following question: “Looking to the future, do you think the current coronavirus emergency will have a positive or negative impact? (a) on your family’s income (b) on your job. For each item, five alternative answers have been provided in the questionnaire: (1) Very negative, (2) Somewhat negative, (3) No change, (4) Somewhat positive, and (5) Very positive.

Thus, we create a unique variable with four categories:

- “Neutral or positive”, whether the respondents’ answer was (3), (4) or (5) for both the items (a) and (b).
- “Negative for me”, whether the respondents’ answer was (3), (4) or (5) for item (a) and (1) or (2) for item (b).
- “Negative for my family”, whether the respondents’ answer (1) or (2) for item (a) and (3), (4) or (5) for item (b)
- “Negative for me and my family”, whether the respondents’ answer was (1) or (2) for both the items (a) and (b)

The following control variables have been also included.

*Gender*: by distinguishing among males and females, we consider that the trajectories characterizing the transition to adulthood may differ by gender.

*Age*: we grouped the individuals in three age classes: 18–24, 25–29 and 30–34.

*Education*: we distinguish young adults who achieved a tertiary level of education (bachelor, master or PhD) from those that achieved an upper secondary education (with a 4- or 5-year high school diploma) and from a residual group including those that have lower levels of education (lower secondary or primary).

*Country*: the country dummies have been introduced to consider the economical and institutional unobserved heterogeneity across countries.

A final issue concerning the choice of the model is the question of selectivity, related to the fact that the analyses are conducted on the subsample of those who expressed a positive intention of going to live on their own. Considering the descriptive aim of this study, a provisional analysis on the distribution of the variables related with the key explanatory variables does not show large differences between the full sample and the subset used in the analysis.

## Empirical results

This section reports the empirical results in the case of parallel lines assumptions are satisfied or less. Alternative specifications of the model are presented: the first one (Model 1) only includes the socio-demographic variables (gender, age, education, employment status and country of origin), while the second model (Model 2) adds the variable related to the perceived future job/income conditions of the respondents and his family.

Finally, a Model 3 has been introduced to consider the moderating role of the context, where the interactions between the country dummies and the explanatory variables (i.e., the employment status and the perceived future job/income conditions) have been included.

As previously said, a Brant test has been implemented with the result of the violation of parallel line assumption for several variables. This means that for the variables for which the condition does not hold, we have different values of the estimates of coefficients for each of the cumulative logit models which have replaced the original ordered one. Specifically, the original ordinal variable has been collapsed into two categories, and a couple of binary logistic regressions are estimated. The first one contrasts category 1 (confirmation of the plan) with a joint category for levels 2 and 3 (postponement or abandonment). The second one contrasts categories 1 and 2, which are collapsed in a unique category, against category 3. Results are reported in Table 4.

**Table 4:** Results of generalized ordered logistic regression for the determinants of the decision of postponing or abandoning the plan of going to live on their own

	Model 1		Model 2		Model 3	
Explanatory variables	Coeff. 3, 2 vs. 1	Coeff. 3, vs. 1, 2	Coeff. 3, 2 vs. 1	Coeff. 3, vs. 1, 2	Coeff. 3, 2 vs. 1	Coeff. 3, vs. 1, 2
**Gender (ref. male)**						
Female	0.032	0.032	0.148	0.148	− 0.048	0.237**
**Age (ref. 25–29)**						
18–24	− 0.083	− 0.083	− 0.074	− 0.074	− 0.043	− 0.043
30–34	− 0.002	− 0.002	− 0.001	− 0.001	0.002	0.002
**Education (ref. tertiary)**						
Upper secondary	0.217**	0.217**	0.223**	0.223**	0.210**	0.210**
< Upper secondary	− 0.286	0.252	− 0.302*	0.310*	− 0.275	0.307*
**Employment (ref. self-employed)**						
Neither student nor employed (NEET)	0.171	0.171	0.152	0.152	0.301	0.301
Student	− 0.127	0.243	− 0.127	0.243	0.513**	0.513**
Permanent employee	0.059	0.059	0.085	0.085	0.298	0.298
Temporary worker	0.265	0.265	0.261	0.261	0.542**	0.542**
**Country of residence (ref: Italy)**						
UK	− 0.170	− 0.170	− 0.164	− 0.164	0.591	0.591
Germany	− 0.635***	− 0.635***	− 0.589***	− 0.589**	0.005	0.005
France	− 0.598***	− 1.453***	− 0.593***	− 1.376***	0.806	0.806
Spain	0.226	− 0.341**	0.240	− 0.320**	0.389	0.389
**Perceived future job/income conditions (ref: positive or neutral for me and my family)**						
Negative for my family but not for me			− 0.082	− 0.082	0.108	0.108
Negative for me only			0.285*	0.285*	0.478*	0.478*
Negative for me and my family			0.432***	0.432***	0.615***	0.615***
**Perceived future job/income conditions × Country**						
Negative for my family but not for me × UK					− 0.377	− 0.377
Negative for my family but not for me × Germany					− 0.227	− 0.227
Negative for my family but not for me × France					− 0.522	− 0.522
Negative for my family but not for me × Spain					1.298	− 1.209*
Negative for me only × UK					0.680	0.680
Negative for me only × Germany					− 1.285***	− 1.285***
Negative for me only × France					− 0.275	− 0.275
Negative for me only × Spain					− 0.146	− 0.146
Negative for me and my family × UK					0.020	0.020
Negative for me and my family × Germany					− 0.649*	− 0.649*
Negative for me and my family × France					− 0.889***	− 0.889***
Negative for me and my family × Spain					− 0.618**	− 0.618**
**Employment × Country**						
NEET × UK					− 0.542	− 0.542
NEET × Germany					0.130	0.130
NEET × France					− 0.885	− 0.885
NEET × Spain					− 0.286	− 0.286
Student × UK					− 1.928***	− 1.928***
Student × Germany					− 1.127**	− 1.127**
Student × France					− 1.287**	− 1.287**
Student × Spain					− 0.063	− 0.063
Permanent empoloyee × UK					− 0.476	− 0.476
Permanent employee × Germany					− 0.225	− 0.225
Permanent empoloyee × France					− 1.195**	− 1.195**
Permanent empoloyee × Spain					− 0.350	− 0.350
Temporary worker × UK					− 1.044*	− 1.044*
Temporary worker × Germany					− 0.125	− 0.125
Temporary worker × France					− 1.214**	− 1.214**
Temporary worker × Spain					− 0.413	− 0.413
*Observations*	*1846*		*1846*		*1846*	

*** indicates a significance at 0.01 level, ** indicates a significance at 0.05 level, * indicates a significance at 0.10 level

By looking at the results from Models 1 and 2, Italy, Spain and the UK are the countries showing the highest probability of postponing or abandoning the purpose of leaving the parental home, while young people living in Germany and France are more likely to confirm the intention of going to live on their own. The violation of parallel line condition for the coefficient of Spain allows to emphasize a specific pattern of preference: Spanish young adults are not significantly different from Italian ones when we compare the decision of confirming the purpose of leaving home with the alternative of delaying or abandoning the plan. However, when we split the alternative of abandoning from that of delaying and we contrast abandoning vs. delay/confirmation the results change, showing Spaniards being less likely to abandon their purpose and generally more optimistic than Italians.

Perceived future job/income conditions are investigated in Model 2, to capture the connection between respondent’s perceived economic vulnerability and the choice of confirming their future plans. We found that how the individual perceives future income conditions is precondition for the revision of the intentions to leave the parental home. In particular, those with negative expectations on their personal future income and on both the personal and family’s future income experience a higher probability of revising their intentions of leaving the parental home.

Model 3 includes the interactions between the country dummies and the main covariates (i.e., the employment status and the perceptions about future income). Only in this case, some coefficients about the occupational conditions come out as significant. In particular, across countries, students and temporary workers are the categories that show higher degrees of vulnerability concerning the future plans. Results from students can be associated with their higher financial dependence from the family of origins, and the concomitant reduction in the chances to move for studying elsewhere during the pandemic. About temporary workers, because of their precarious occupational condition, their higher vulnerability in the labour market—compared with the other working categories—in times of crisis might be associated with their higher likelihood of postponing or abandoning the choice of leaving the parental home.

As we can see from the last two columns in Table 4, the main effects of the country dummies lose their significance when their interactions with the main covariates have been introduced. If we look at the interactions between the country dummies and the occupational conditions, these associations vary across countries. In Italy, Spain and Germany, temporary workers show higher probability to postpone or abandon the pre-COVID plan compared with those in the UK and France. Regarding the student condition, in Italy and Spain, students are more prone to change their plans than in the UK, Germany and France. This might be linked to the fact that while in some countries, and especially in the UK, students in higher education are encouraged to leave the parental home and attend universities with on-campus accommodation, in other contexts, such as Italy or Spain, the widespread presence of universities within the country allows students not to move away from home.

Main results about the perceived future job/income conditions confirm a significant and moderate association between occupational and economic vulnerability of the individual and the decision of abandoning/postponing the plan of leaving the parental home. Coherently, a pessimistic point of view involving both the individual and the familiar dimension strengthens the effect. If we consider the moderator effect of the country of residence—compared with Italy and the UK—Germany, France and, to a lower extent, Spain display a reduced effect of the perceived future economic condition on the decision of leaving the parental home.

Finally, we provide a comment about the control variables, which violate the parallel line assumptions with respect to the role played by gender (in Model 3) and education (in all the models), confirming the goodness of the choice of a generalized logistic regression instead of an ordinal one. Women are not more likely to change their plans compared with men (in Models 1 and 2). In Model 3, after having interacted the main predictors with the country dummies, the parallel line assumption is violated: women seem to be more likely to abandon their plans, when the choice of abandoning is contrasted to that of confirming or postponing the plan. This element suggests the need of focusing on the fragility of the women’s condition in the labour market. The effect of education is also interesting, especially referring to Model 2. While individuals with a secondary education show a higher probability of delaying or abandoning the intentions of departing from the family of origin, lower educated individuals are less likely to opt for a delay compared with higher educated ones. In the model contrasting the choice of confirming to postponing/abandoning, the coefficient is negative, indicating that lower educated young adults are more likely to confirm. When we contrast the choice of confirming or delaying the plans versus the choice of abandoning the plan, the coefficient changes the sign and become positive, indicating a preference for renouncing to the purpose of exiting from the parental home. In both the cases, the lower educated individuals seem to polarize their preferences toward the two opposite alternatives: leaving the parental home even in presence of the COVID emergency or totally abandoning the plan.

## Robustness checks

To confirm the goodness of the estimates, some robustness checks have been performed (results are in the Appendix).

In the first check, we run generalized logistic models introducing the youth unemployment rate among the explanatory variables: the variable measures the youth unemployment in 2019 at the regional level (NUTS1 or NUTS2 according to the minimum level of aggregation allowed in the Youth Report data. Source: Eurostat database), and it represents an indicator of the presence of a favourable or unfavourable labour market. In the first case (Model 4 of Table 5 in Appendix), the youth unemployment rate is alternative to the use of geographical area dummies, and its coefficient is significant and negatively related to the choice of confirming the pre-COVID plan of leaving the parental home. Nevertheless, youth unemployment may be not the equivalent of the set of the country dummies, which can also capture unobserved heterogeneity at country level that goes beyond the macroeconomic effect of labour market condition. Hence, Model 5 includes both the youth unemployment rate and the country dummies. As expected, the youth unemployment rate loses its significance, while the country effects remain generally close to those observed in Model 2 of Table 4. This suggests that the country dummies absorb both the effects of the labour market and the residual unobserved heterogeneity at country level. This result is confirmed also after introducing the interactions of the main covariates, as it emerges in the comparison between Model 6 of Table 5 in Appendix and Model 3 of Table 4.

Finally, results of standard-ordered logits are also displayed in Table 6 in Appendix. Although the model gives less consistent estimates, results are in line with those from the main analyses.

## Conclusions

This study offers an explorative and descriptive analysis of the possible effect of the COVID-19 pandemic on the young population projects of leaving the parental home and going to live on their own, by comparing a set of European countries. As far as we know, this is the first study on the topic conducted on international representative samples of the young population (18–34) of five European countries (from data on the same survey, see also Luppi, Arpino, & Rosina, 2020). Results from our paper suggest that precarious employment situation and bad perspective financial conditions of the young individuals and their families are associated with a negative revision of their intention of leaving the parental home because of the COVID-19-related economic crisis. This path is particularly stressed in Italy and Spain, and partially in the UK. Here, having a temporary job (in Italy and Spain) and feeling insecure about the future financial situation (also in the UK) are strong predictors of a downward revision of the original plan about reaching housing autonomy. These results seem to confirm the idea that both the objective conditions of the present and the perceived vulnerability about the future matter for the life plans of young people during the COVID-19 pandemic.

In particular, the vulnerability of temporary workers needs to be taken into serious consideration by policy makers, especially because this condition is dominant for those entering in the labour market (Ashton, 2017; Bloodworth, 2018; Standing, 2011). Fixed-term, temporary and 0-h contracts are particularly common among the youngest, 16- to 24-year-olds (Eurostat,^ 2017^), and in countries such as Spain, Italy and Germany. However, while in Germany temporary contracts are often transitory to permanent contracts, in Italy and Spain, temporary contracts are involuntary in most of the cases and they less frequently end up into permanent jobs (Mascherini, Ledermaier, Vacas-Soriano, & Jacobs, 2017). In the UK, temporary contracts are much rarer than in other European countries, and in one third of the cases, they are adopted even by worker’s choice (Schoon & Bynner, 2019). This mirrors the liberal soul of the UK economy and the higher level of de-regularization of the labour market, where dismissing is much easier than elsewhere.

Across countries, the propensity to revise the original plan of attaining a residential autonomy remains higher in Italy, Spain and the UK, even controlling for the occupational condition and the expected effect of the crisis on the individual’s and family’s income. As argued, the institutional framework plays a primary role in shaping the opportunities and the resources that young people can access to support their transition towards the autonomy from the family of origin. The uncertainty brought by the COVID-19 health and economic crisis can only magnify the contextual effect on this process. Therefore, familialistic as liberal welfare regimes seem to not offer enough financial guarantees to support the decision to leave the nest during period of great (global) uncertainty. At the same time, previous unfavourable conditions of both the economic system and the labour market do not provide young people with a solid ground on which they can build their own house. Even though we still do not have evidence on how the current pandemic and economic crisis will affect future concrete behaviours, results from our study suggests a negative impact of the current recession at least on the intentions of the young generations to pursue their life plans.

Our study is not without limits. The small sample size does not allow to further explore other possible moderator or mediation effects. Moreover, because the survey has been conducted at the beginning of the health emergency, our results mirror the very first shock caused by the first lockdown; therefore, the enduring effect or a possible adaptation to the crisis, which might be evident only late in 2020, have not been detected. At the beginning of the health emergency, in fact, people were not fully conscious about the duration and the strength of the crisis: the newspapers said that the pandemic was spreading and worsening, leading to a severe economic recession, but at that time basically no one knew how long the crisis would have lasted and how deep the recession would have been. However, the second pandemic wave, which occurred just after the summer break, has shown how far we were from the end of the emergency. The start of the 2021 vaccination campaign represented the first sign of a possible close U-turn of the trend, even though the third wave of the epidemic and the spread of the COVID-19 variants are still fostering people’s uncertainties about the end of the crisis.

Hence, a follow-up of this study should be implemented to evaluate the enduring effect of the COVID crisis on the young people’s plan to gain a residential autonomy from the family of origin. Further waves of the same survey may also explore the eventuality of those individuals who did not plan to leave the parental home in January 2020, but they still moved to live alone during the pandemic. Finally, because of data limitations, the heterogeneity of the reasons for leaving the parental home cannot be fully controlled for. This implies a possible selection of the individuals with different propensity to exit the parental home across countries.

Therefore, further studies are needed to estimate the effect of the current crisis on the actual realization of the young generation’s life plans in the next future. Still, our first empirical evidence suggests urgent policy interventions to support young people in getting their economic and housing independence, especially in those countries where the transition to adulthood was already an issue before the pandemic occurred.

## Data Availability

The data that support the findings of this study are available from Istituto Toniolo, but restrictions apply to the availability of these data, which were used under license for the current study, and so are not publicly available. Data are however available from the authors upon reasonable request and with permission of Istituto Toniolo.

## Appendix

**Table 5:** Results of generalized ordered logistic regression for the determinants of the decision of postponing or abandoning the plan of going to live on their own

	Model 4		Model 5		Model 6	
Explanatory variables	Coeff. 3, 2 vs. 1	Coeff. 3, vs. 1, 2	Coeff. 3, 2 vs. 1	Coeff. 3, vs. 1, 2	Coeff. 3, 2 vs. 1	Coeff. 3, vs. 1, 2
**Gender (ref. male)**						
Female	0.106	0.106	0.150	0.150	− 0.046	0.239**
**Age (ref. 25–29)**						
18–24	− 0.082	− -0.082	− -0.069	− 0.069	− -0.040	− 0.040
30–-34	0.009	0.009	− 0.001	− -0.001	0.002	0.002
**Education (ref. tertiary)**						
Upper secondary	0.242**	0.242**	0.224**	0.223**	0.211**	0.211**
< Upper secondary	− -0.336**	0.400**	− -0.302*	0.311*	− 0.275	0.308*
**Occupational status (ref. self-employed)**						
Neither student nor employed (NEET)	0.049	0.049	0.147	0.147	0.291	0.291
Student	− 0.166	0.165	− 0.131	0.239	0507**	0507**
Permanent employee	0.065	0.065	0.089	0.089	0.305	0.305
Temporary worker	0.223	0.223	0.259	0.259	0.539**	0.539**
**Country of residence (ref: Italy)**						
UK			− 0.101	− 0.101	0.642	0.642
Germany			− 0.516***	− 0.516***	0.063	0.063
France			− 0.555***	− 1.337***	0.834	0.083
Spain			0.239	− 0.320**	0.387	0.387
**Perceived future job/income conditions (ref: positive or neutral for me and my family)**						
Negative for my family but not for me	− 0.016	− 0.016	− 0.079	− 0.079	0.115	0.115
Negative for me only	0.306*	0.306*	0.283*	0.283*	0.476*	0.476*
Negative for me and my family	0.181	0.552***	0.090	0.433***	0.618***	0.618***
**Perceived future job/income conditions × country**						
Negative for my family but not for me × UK					− 0.384	− 0.384
Negative for my family but not for me × Germany					− 0.233	− 0.233
Negative for my family but not for me × France					− 0.529	− 0.529
Negative for my family but not for me × Spain					− 0.139	− 1.216*
Negative for me only × UK					0.648	0.648
Negative for me only × Germany					− 1.282***	− 1.282***
Negative for me only × France					− 0.273	− 0.273
Negative for me only × Spain					− 0.139	− 0.139
Negative for me and my family × UK					0.018	0.018
Negative for me and my family × Germany					− 0.651*	− 0.651*
Negative for me and my family × France					− 0.890***	− 0.890***
Negative for me and my family × Spain					− 0.616**	− 0.616**
**Occupation × country**						
NEET × UK					− 0.533	− 0.533
NEET × Germany					0.139	0.139
NEET × France					− 0.876	− 0.876
NEET × Spain					− 0.280	− 0.280
Student × UK					− 1.925***	− 1.925***
Student × Germany					− 1.122**	− 1.122**
Student × France					− 1.282**	− 1.282**
Student × Spain					− 0.062	− 0.062
Permanent empoloyee × UK					− 0.483	− 0.483
Permanent empoloyee × Germany					− 0.230	− 0.230
Permanent empoloyee × France					− 1.200**	− 1.200**
Permanent empoloyee × Spain					− 0.352	− 0.352
Temporary worker × UK					− 1.044	− 1.044
Temporary worker × Germany					− 0.123	− 0.123
Temporary worker × France					− 1.210**	− 1.210**
Temporary worker × Spain					− 0.412	− 0.412
**Youth unemployment rate**	0.016***	0.016***	0.003	0.003	0.003	0.003
*Observations*	*1846*		*1846*		*1846*	

*** indicates a significance at 0.01 level, ** indicates a significance at 0.05 level, * indicates a significance at 0.10 level

**Table 6:** Results of ordered logistic regression for the determinants of the decision of postponing or abandoning the plan of going to live on their own.

	Model 7	Model 8	Model 9	Model 10	Model 11
Explanatory variables	Coeff.	Coeff.	Coeff.	Coeff.	Coeff.
**Gender (ref. male)**					
Female	0.183**	0.149	0.109	0.107	0.109
**Age (ref. 25–29)**					
18–24	− 0.084	− 0.066	− 0.077	− 0.039	− 0.036
30–34	− 0.007	− 0.003	0.009	0.001	− 0.001
**Education (ref. tertiary)**					
Upper secondary	0.211**	0.214**	0.232**	0.203**	0.204**
< Upper secondary	− 0.039	− 0.014	0.016	0.003	0.003
**Occupational status (ref. self-employed)**					
Neither student nor employed (NEET)	0.190	0.166	0.056	0.329	0.329
Student	0.073	0.071	0.009	0.544**	0.539**
Permanent employee	0.081	0.105	0.073	0.324	0.331
Temporary worker	0.280*	0.269	0.219	0.572**	0.569**
**Country of residence (ref: Italy)**					
UK	− 0.202	− 0.203		0.601	0.653
Germany	− 0.708***	− 0.656***		− 0.040	0.018
France	− 0.920***	− 0.883***		0.433	0.462
Spain	− 0.115	− 0.107		0.426	0.423
**Perceived future job/income conditions (ref: positive or neutral for me and my family)**					
Negative for my family but not for me		− 0.086		0.104	0.105
Negative for me only		0.257*		0.496*	0.496*
Negative for me and my family		0.282**		0.649***	0.649***
**Perceived future job/income conditions × country**					
Negative for my family but not for me × UK				− 0.361	− 0.368
Negative for my family but not for me × Germany				− 0.207	− 0.212
Negative for my family but not for me × France				− 0.468	− 0.475
Negative for my family but not for me × Spain				− 0.203	− 0.211
Negative for me only × UK				0.696	0.696
Negative for me only × Germany				− 1.386***	− 1.384***
Negative for me only × France				− 0.345	− 0.342
Negative for me only × Spain				− 0.161	− 0.154
Negative for me and my family × UK				0.007	0.005
Negative for me and my family × Germany				− 0.679*	− 0.680*
Negative for me and my family × France				− 0.882***	− 0.883***
Negative for me and my family × Spain				− 0.632**	− 0.631**
**Occupation × country**					
NEET × UK				− 0.571	− 0.561
NEET × Germany				0.153	0.162
NEET × France				− 0.818	− 0.808
NEET × Spain				− 0.305	− 0.297
Student × UK				− 1.978***	− 1.975***
Student × Germany				− 1.122**	− 1.117*
Student × France				− 1.170**	− 1.166**
Student × Spain				− 0.138	− 0.136
Permanent empoloyee × UK				− 0.485	− 0.491
Permanent empoloyee × Germany				− 0.232	− 0.237
Permanent empoloyee × France				− 1.042**	− 1.047**
Permanent empoloyee × Spain				− 0.356	− 0.356
Temporary worker × UK				− 1.073*	− 1.073*
Temporary worker × Germany				− 0.071	− 0.068
Temporary worker × France				− 1.101**	− 1.098**
Temporary worker × Spain				− 0.463	− 0.463
**Youth unemployment rate**			0.017***		0.003
*Observations*	*1846*	*1846*	*1846*	*1846*	*1846*

*** indicates a significance at 0.01 level, ** indicates a significance at 0.05 level, * indicates a significance at 0.10 level

**Table 7:** Sample details as collected in the Youth project official statement (Istituto Toniolo, 2021)

	Total of young adults aged 18-34^**a**^	Sample size	Statistical margin of error	
**Country**				
Italy	10,630,814	2000	± 0.4	± 2.2
France	13,226,017	1000	± 0.6	± 3.1
UK	14,659,036	1000	± 0.6	± 3.1
Germany	16,906,500	1000	± 0.6	± 3.1
Spain	8,540,984	1000	± 0.6	± 3.1

^a^Istat/Eurostat, data at 1st of January 2019

## Footnotes

1 The results of the Brant test are performed for each coefficient in every model. Hence, they are omitted from the table. When the test result leads to reject the parallel line assumption for a single coefficient, its estimate is different when the cut point of the dependent variable changes. Otherwise, the coefficient is the same, even if the cut point of the cumulative logit varies.
